# Early prognostic indices for weaning after long-term mechanical ventilation

**DOI:** 10.1186/cc9597

**Published:** 2011-03-11

**Authors:** A Temelkov, R Marinova, M Lazarov

**Affiliations:** 1 Alexandrovska University Hospital, Sofia, Bulgaria

## Introduction

A large number of predictive indices are used for evaluation of the capability for transition to spontaneous breathing in critically ill, mechanically ventilated patients. The great number of these indices and the difficulties in the interpretation causes significant obstacles and unclear points during the early attempts for transition to spontaneous breathing. In our study we investigated the role of predictive indices that are significant for weaning after long-term mechanical ventilation. The purpose is to determine predictive indices, which have early and significant predictive value concerning successful transition to spontaneous breathing.

## Methods

The study covers 45 critically ill patients who were mechanically ventilated for more than 7 days in our ICU. The weaning efforts were made through a T-circuit for spontaneous breathing according to the local protocol. The patients were allocated into two groups - group A (38 patients with successful 2-hour spontaneous breathing through a T-circuit) and group B (seven patients with unsuccessful 2-hour test of weaning with a T-circuit system). The monitored parameters in this period were: respiratory rate/tidal volume ratio (f/Vt), occlusive pressure (Po.1), inspiratory time/tidal time ratio (Ti/Ttot), pressure time index, pressure time product and work of breathing (WOBp) together with SAPS II score and clinical and paraclinical parameters, concerning successful weaning.

## Results

Clinical research of f/Vt and WOBp between the two groups gives a reliable index in transition to spontaneous breathing. Changes in Po.1, Ti/Ttot, pressure time index and pressure time product are later and thus less important in the early assessment of withdrawal after long-term mechanical ventilation.

## Conclusions

Respiratory rate/tidal volume ratio (f/Vt) and work of breathing (WOBp) are the earliest predictive indices for the possible outcome in the process of weaning after long-term mechanical ventilation.
